# Sense of Coherence, Compassionate Love and Coping in International Leaders during the Transition into the Fourth Industrial Revolution

**DOI:** 10.3390/ijerph17082829

**Published:** 2020-04-20

**Authors:** Claude-Hélène Mayer, Rudolf M. Oosthuizen

**Affiliations:** 1 Department of Industrial and Organisational Psychology, University of Johannesburg, Johannesburg 2006, South Africa; claudemayer@gmx.net; 2 Institut für therapeutische Kommunikation und Sprachgebrauch, European University Viadrina, 15230 Frankfurt (Oder), Germany; 3 Department of Industrial and Organisational Psychology, University of South Africa, Pretoria 0002, South Africa

**Keywords:** salutogenesis, sense of coherence (SOC), compassionate love (CL), leadership, coping (C), 4IR workplaces, societal and cultural challenges

## Abstract

Contemporary workplaces are influenced by rapid changes, high levels of competition, increasing complexities and internationalisation processes. At the edge of the Fourth Industrial Revolution (4IR), insecurities and anxieties are high, and leaders are encouraged more than ever to lead employees with meaningful vision and prudence in order to make use of employees’ strengths, and ensure mental health and well-being. The aim of this article is to present new insights into salutogenesis, particularly sense of coherence (SOC), compassionate love (CL), and coping (C) in leaders with different cultural backgrounds. This study strengthens the idea that CL is a coping mechanism. This coping mechanism can be used by leaders to establish a resilient and salutogenic organisations. This article explores the subjective perspectives of 22 international leaders from five different countries and their views regarding SOC, CL and C through a qualitative research approach, using a qualitative online questionnaire for data collection and content analysis for data analysis. The findings on the perspectives of leaders provide new and original insights into how SOC, CL and C are connected, and how these concepts contribute to healthy organisations which are on their way to the transition into the 4IR. Conclusions are drawn. Recommendations for future research and practice are given.

## 1. Introduction

The Fourth Industrial Revolution (4IR) is characterised by rapid changes on socio-economic, political and cultural levels. It brings increasing technology-based human–machine interaction, growing digitalisation, and increasing use of smart technologies. These changes also bring about a change in workplaces cultures [1]. Individuals are challenged with understanding complexities, managing them constructively and redefining the meaning of work [2]. Rapid changes, however, cause stress and negative emotions, such as anxiety, frustration and sadness, as well as disorientation, insecurities and ambiguities [3]. Leaders are encouraged to provide guidance and leadership to address new challenges and support employees to cope positively and constructively [4]. Positive emotions in leadership can support salutogenesis and coping [5].

Salutogenesis is concerned with developing, maintaining and increasing the health of individuals [6,7,8]. The main construct of salutogenesis, sense of coherence (SOC), is a resource and life orientation which supports individuals as a coping mechanism and fosters health, even in stressful situations [9]. SOC consists of three components [6] that will be explored in this study: comprehensibility, manageability and meaningfulness.

Coping (C) can be portrayed as the capacity with which leaders handle an unpleasant occasion [10]. Leaders perceive SOC as a useful and functional guide to behaviour, which gives SOC a prominent role in C in the 4IR context. SOC directs leaders to focus on a specific set of stimuli out of all the possible stimuli with which they could possibly cope. By emphasising important elements of the environment, emotions motivate cognitive and behavioural reactions. Leaders are only likely to perceive a 4IR challenge when emotions are evoked at the same time. Additionally, SOC and compassionate love (CL) prepare leaders for action in response to stimuli [11,12].

The concept of compassionate love (CL) has gained interest in leadership studies [13], highlighting that the concept impacts positively on empowerment, authenticity, and stewardship, providing direction to employees [14]. Compassionate love also increases empathy for others in addition to authentic listening, nurturance and caring skills [15,16,17]. CL embodies and enacts the qualities of love, altruism, integrity, humility and wisdom combined with an appreciation and empowerment of others [18]. CL is core to the development of a compassionate and person-centred organisation and requires senior leaders to clearly articulate the core values and vision of the organisation and to ensure that they resonate in all the self-organising groups within the system [18]. CL supports leaders by helping them cope with job demands, manage stress and conflicts, set pro-social goals and connect with their employees and stakeholders [17].

The findings of Lloyd [19] indicate that higher levels of CL and SOC were both associated with lower stress. The findings also indicate that CL is positively associated with adaptive C strategies, and both CL and SOC are negatively associated with avoidance-oriented strategies. SOC and maladaptive C emerged as significant predictors of perceived stress in subsequent regression analyses. Interventions or support mechanisms that enhance SOC and reduce reliance on maladaptive C may decrease vulnerability to stress in leaders.

### 1.1. Aim of This Article

The aim of this article is to present the perspectives of leaders on SOC, CL and C and interrelationships that exist therein with regard to their professional work and leadership during transition into the 4IR in different cultural contexts.

Since the literature on the 4IR often focuses on the negative side—technological challenges, fears and rapid, incomprehensible complexities—this study aims to focus on positive aspects in terms of how leaders across the globe cope with these challenges. Leaders were asked about SOC, C and CL in terms of the transition into the 4IR. The leading research question for the findings presented here is: how do SOC and CL support C in leaders during transition into the 4IR? The contribution of this study is to investigate SOC and CL in relation to C, and thereby close a void in the literature.

The relationship between SOC and C is well established in the literature, as well as between self-compassion, SOC, and C strategies, but SOC and CL have not been investigated in relation to C in leaders. Thus, the authors will present the core concepts of this study in more depth.

### 1.2. The Transition into the Fourth Industrial Revolution (4IR)

Leaders face workplace changes and rapid development, increased technologisation, digitalisation, smart-system use, artificial intelligence, questions about the meaning of work, innovation and creativity in 4IR contexts [20,21]. These changes pose challenges to organisations and societies [22]. Magubane [23] suggests that love needs to be part of the transition into the 4IR. Illouz [24,25] sees changes regarding love within the 4IR taking on modern shapes, such as alternate conceptual and relationship changes that are mediated through technological interactions or robotics.

Chandsoda and Salsing [26] emphasise that the 4IR requires people to incorporate sympathy and participation into human relations, work environments and leadership. Van der Hoven [27] emphasises that individuals ought to re-learn how to detach from the advanced world and how to reconnect compassionately with self, others and nature. CL means reconnecting with one’s “true self” and nature, collaborating with others, disengaging from the computerised world, tuning into one’s inward voice and achieving a state of flow and imagination [27]. Tu and Thien [28] recommend that the fundamental standards of Buddhism offer assistance to leaders through the 4IR, by promoting mindfulness, contemplation, love and trust as basic values.

Sun [29] suggests that organisations ought to utilise “love competition”. “Love competition” is a competitive attitude based on CL, shrewdness and market-orientated social skills. Fabritius [30] contends that stress levels, potential conflict, and work insecurity, alluding to “fear of unemployment and trouble of reemployment,” are rising [31,32]. Different trends in the 4IR, such as instable working conditions, automation, and fear of loss of work lead to poor health and negative feelings, such as anxiety [33,34,35]. How do leaders in different cultural contexts cope with the transition into the 4IR?

### 1.3. Salutogenesis, Sense of Coherence and Leadership

Leaders increasingly focus on promoting health and well-being in employees in order to improve the performance of organisations [36]. Salutogenesis has become a favourable approach to increasing health and well-being (source), exploring factors affecting and implementing programmes supporting employee health [37,38,39,40,41,42]. According to Antonovsky [6], health and well-being are developed through SOC. SOC is a global life orientation that supports individuals in comprehensibility, manageability and meaningfulness—leading to consistent congruence and harmony within an individual. The more pronounced an individual’s SOC, the healthier an individual is [43].

Comprehensibility refers to one’s understanding of the world based on the ability to process familiar and unfamiliar stimuli as ordered, structured and consistent; manageability, relates to how one copes with challenges and whether one believes that challenges can be solved through the use of resources; meaningfulness relates to how an individual is motivated through the construction of meaning in life and the extent to which life makes sense [7].

SOC supports individuals in focusing on their C strategies and helps leaders to stay healthy in stressful situations [42,44]. SOC prevents mental illness [45] and helps individuals to cope within complex, international and intercultural settings [46]. A high SOC impacts positively on performance, achievement, success and the ability to manage conflicts and intercultural communication [43].

Individuals with a high SOC are more likely to perceive the leader as a good listener, and as an engaging and encouraging intuitive thinker. They also include employees in decision-making and problem-solving and foster participation. This, in turn, leads to employees who are energetic, enthusiastic, proud, inspired and happily engaged in their work [46,47]. Compassionate leadership impacts positively on employees, encouraging creativity in problem solving and a high level of engagement.

Organisations that advance and improve working conditions in terms of physical, mental and social well-being, are more likely to have employees perceiving their leaders’ management style as positive (i.e., manages who are supportive, consider their concerns, empower them, and listen, including co-operating with them and providing counselling) [46,47,48]. These employees demonstrate high levels of psychological connection, engagement and performance [47].

SOC can be applied at different system levels, including at the individual, group, organisational and societal levels [49], and is a source of compassionate leadership that supports the development of a strong SOC [50]. Recent leadership research shows that love—defined as a complex concept of compassion, humanity, care and unconditional, compassionate love—impacts positively on leaders in terms of coping with work challenges, such as changes faced during the transition into the 4IR. Harry [51] provides insights, showing the relationship between individuals’ wellness attributes (sense of coherence, emotional intelligence and burnout) and their resiliency capacities (career adaptability and hardiness). Organisations need to understand the complex processes involved in coping during transition into the 4IR [52] and the importance of fostering SOC.

### 1.4. Coping and Compassionate Love in Leaders

Leaders could potentially perceive a 4IR challenge, appraise it as such and cope with it without necessarily experiencing intense feelings or emotions. C with emotions includes the idea of C with the leaders’ own emotions beyond emotion regulation. Emotion regulation includes conscious efforts to modulate the intensity of leaders’ emotions (for example by avoiding emotion-eliciting 4IR situations or by reappraising the meaning of a 4IR situation) [53]. In contrast, C with emotions can include behaviours elicited by an emotion, regardless of whether the behaviours are intended to regulate the emotional experience [54].

Leaders respond differently to emotions [54,55]. The differences in how leaders cope with emotional states can have implications for their long-term psychological and physical health [54]. Machin, Adkins, Crosby, Farrell, and Mirabito [56] postulate that C efforts are actions taken to protect, maintain, or restore well-being. Future-focused and action-oriented challenges promote anticipatory and preventative C strategies. Rather than reacting to immediate 4IR stressors, future-oriented C strategies instead seek to protect the leader before harm occurs. Understanding the goal behind a C tactic also helps in judging coping effectiveness, a troubling issue in contemporary research [57].

Love has been ignored in Western writing on leadership for a long time [58]. However, Barsade and Gibson [59] mention the exceptional role of CL in working environments: CL affects the demeanour of employees, their accomplishments, the organisational culture and the employee relationships. Positive feelings have an immense and positive impact on employees and their performance [60,61,62]. Several researchers [63,64] have indicated that compassionate love is associated with care, concern und thriving in connection with others. CL is connected to kindness, affection, open-mindedness, caring and kind-heartedness [63]. CL further energises an attitude of humbleness, gratitude, forgiveness and selflessness in leaders within the work environment [14]. CL needs new research and detailed descriptions in organisational contexts [65].

Van Dierendonck and Patterson [14] describe CL as inspiring, meaningful and optimal social functioning in organizations. CL fosters encouragement, genuineness, stewardship and leadership. Barsade and O’Neill [66] state a universal conviction that employment relationships do not contain adequate deepness to be named “love” relationships. Other researchers counter-argue that social relationships at work transfer in-depth emotive encounters and are full of meaning [67]. Savickas [68] suggests that CL at work can significantly affect careers and happiness at work, while O’Neill [69] suggests that CL is a critical factor in employment relationships, which include caring, compassion and gentleness. Emotions perform an exceptional role at work [66] and CL shields negative emotions, such as fear [70]. CL is often referred to in work contexts with regard to compassionate leaders [71].

Rapport [72] states that love within the work environment can be depicted as a frame of moral engagement which goes past culture and signifies a collective culture of morals. CL is often based on values which cultivate natural inspiration—such as spiritual well-being or a calling—compassion and joy, meaning making and sense making, hope, faith and altruistic love (including care, concern, and appreciation for both organisational and employee needs) [73]. CL leads to the removal of fears related to anger, failure, selfishness, guilt or worry [74] and increases vitality and the feeling of being lively within the working environment [75,76].

CL encourages meaningful and optimal human functioning, impacting on the leader’s propensity towards virtues, such as humility, gratitude, forgiveness and altruism. The salutogenic approach includes the effectiveness of C processes. It concentrates on what differentiates "successful copers," even in the most stressful of situations, from others and, thus, seeks out personality traits and protective factors that are related to successful C in stressful situations. Work within the framework of this approach mainly involves SOC [7]. Leaders comprehensibility could to a large extent be constructed by their own thoughts and theories. Manageability can be achieved by active information-seeking strategies, social support and C, including positive reinterpretation of the 4IR context. Meaningfulness may be central for quality of life and is achieved through CL and close relations, as well as by work. SOC integrates essential parts of the stress/C model (comprehensibility, manageability) and of CL (meaning).

## 2. Research Methodology

### 2.1. Research Paradigm

This study is anchored in the qualitative research paradigm and uses a hermeneutical and interpretative framework for analysis and interpretation [77,78]. Hermeneutics are defined as the philosophy and interpretation of meaning [79], focusing on the meaning of a text or a text analogue [80].

### 2.2. Sampling

During the sampling process, purposeful sampling and snowball sampling were used [81]. The researchers used purposeful sampling to ensure that the sample can relate to the research topics. Participants were asked to respond to the questionnaire and refer to their own experiences with regard to the selected concepts. Information about the participants is given in the Findings section. The sample consisted of 22 individuals—9 females and 13 males. The age ranged between 33 and 80 years. Interviewees referred to their religion/belief as follows: seven were Christians; four Roman Catholics; two Protestants; one each atheist, agnostic, atheist/agnostic, Buddhist, Muslim, Jew, and Jesuit; one indicated no religious affiliation.

The sample was classed as “international” due to the fact that leaders came from various countries, and national and cultural backgrounds and all acted in international projects, cooperations and networks. Ten of the participants spoke German as their first language, five English, two Afrikaans and one participant each spoke Japanese, Tswana, Hebrew and Romanian. In terms of nationality, the sample consisted of eight Germans, five US citizens, two Japanese, two South Africans, and one participant each with White, German-Iranian, Israeli, Romanian and Bavarian origin (Bundesland (provincial state) in Germany). In terms of educational background, the participants held the foillowing degrees: nine doctoral degrees, six master’s degrees, three “Diploma” degrees (Diplom degrees are equivalent to master’s degrees in Germany), two post-doctoral degrees, one national diploma, and one high school certificate. The sample further included seven professors, three academics, three directors, two consultants, one professor/entrepreneur, one entrepreneur, one executive manager, one project manager, one teacher, one psychologist, and one educational officer. All of the leaders held leadership positions in educational fields and had held them for at least two years. All leaders find themselves at “the edge of the 4IR” as organisations in which the leaders’ work aim towards a transition into the 4IR using digitalisation and smart systems. Some parts of the work are automated and several organisations are starting to collect and analyse big data—all signs of a transition into the 4IR.

### 2.3. Data Collection and Analysis

Data were collected through structured questionnaires [82] that were sent out to the participants via email. Participants filled in the online questionnaires and sent the filled-in questionnaires back to the researcher. The questionnaires included questions referring to sense of coherence, comprehensibility, manageability and meaningfulness, coping, leadership and compassionate love (see Appendix A). The interviewees indicated the lengths of time they took to fill in the questionnaires. The time ranged from 90 to 240 min.

Data were analysed through the five-step process of content analysis by Terre Blanche, Durrheim, and Painter [83]: (1) familiarisation and immersion, (2) inducing themes, (3) coding, (4) elaboration, and (5) interpretation and checking to ensure data quality. Interviews were coded and categorised by using a deductive research interpretation process, focusing on the constructs of SOC, CL and C. Researchers used an intersubjective validation approach, comparing and discussing the process and codes [84].

The authors considered the usual quality criteria for qualitative research, such as conformability, credibility, transferability and dependability, as well as rigor [85]. These quality criteria were addressed through all the phases of research. In terms of ethical considerations, all participants participated voluntarily, were informed about this study, its aim and purpose and consented to this study.

### 2.4. Ethical Considerations

Ethical considerations included: open discussion of the rights of the participant, respectful communication, respect, the creation of informed consent, confidentiality, anonymity and transparency [86]. Ethical approval for this research study was given by the Institut for Therapeutic Communication and Language Use, Europa Universität Viadrina, Frankfurt (Oder), Germany.

## 3. Results

In the following section, the findings on sense of coherence, coping and compassionate love in leaders are presented. Direct quotations are only provided regarding the three most frequently mentioned categories, and reference to other categories is only provided by description.

### 3.1. Salutogenesis and Compassionate Love in Leadership

When leaders were asked how compassionate love and salutogenesis are connected, 19 out of 23 leaders responded that compassionate love is the basis for salutogenesis. P10, a Japanese female leader, 59 years, emphasises that compassionate love and salutogenesis are strongly interwoven concepts:

“Yes, they are connected tightly. Mental health and well-being provide us with energy to embrace and love others, and vice versa.”

P15, a female Israeli leader, also recognises the interconnectedness of CL and health:

“People who have true love and are in good relationship, are physically and mentally healthier. They live longer, less depressed, report fewer pains and more happiness.”

Eight participants said that CL is their resource to cope at work. Altogether, seven participants mentioned that compassionate self-love is their core C resource that fosters mental health. P1, a German female leader, emphasises a combined perspective:

“Love is in my view a really crucial factor for happiness, well-being. Although I always used to think that love must be a partner, today I continue to think about it and I enjoy the many loves in my life. …Love is a very important factor for my personal well-being and my health. It is not just about being loved, but above all about loving oneself. In me is an ocean of love, I have to share, otherwise I may drown in myself;-) At the same time, the world needs more love, it is a resource…”

Further the findings (Table 1) show that seven leaders highlight that CL is a universal emotion and part of “being human”. “Being human” is very important in leadership (P1). For six other participants, CL is a general source of happiness and. as such, it is connected to salutogenesis. For five other individuals, CL is the force that creates their inner being, but also the outer balance and harmony they need in their demanding position. CL determines how they feel about people and situations, and how they act and respond. Four individuals highlighted that, if they have an intra-personal feeling of CL towards the world, they are content and happy with their leadership. Happiness further induces salutogenic feelings being at ease with the world. Finally, two individuals view CL as a base for their inner safety and salutogenesis.

In the following section, the findings are presented with regard to the three components of SOC as aspects which promote and foster mental health and well-being when integrated.

### 3.2. How do You Understand the World of Work? (Comprehensibility)

Altogether, 13 leaders say that they can cope best when they understand the world in its complexity and by applying an attitude of CL (Table 2). P19, a Romanian female leader, points out:

“I believe love transcends culture. It is above it and above everything else. Love is a bridge. It is universal, even if expressed differently across cultures. We understand love even if we belong to different cultural backgrounds. This understanding is important to be together and work together. Love is love. We all know when we are being loved. And it helps us to understand each other.”

Further, eight leaders highlight that attitude is not only relevant in terms of comprehensibility but also brings appreciation. P8, a male German-Iranian leader, emphasises:

“Appreciation is a main key to open yourself for other people. If you show appreciation, people will open up and understand that a transcultural faux pas was not an intended insult.”

P9, a German male leader, mentions:

“Love brings about an appreciation for self and others through its positive energy and strengths. When we love, we feel energized and healthy. And when we appreciate the other we might understand him better.”

According to seven leaders, comprehensibility is fostered through a willingness to learn about others. P14, a male South African leader, emphasises:

“In leadership and co-operation, we need to show interest, objectivity, concern, justice, empathy, listening skills, support, willingness to go the extra mile, encouragement, openness, warmth, honesty, a smile, little positive deeds, positive action, word of honour. We need to stay willing to learn, be open. Then cooperation across cultures will work out. But probably it is not valued enough.”

Finally, listening with patience and kindness—aspects which are described as part of CL—brings a deeper comprehensibility of complex, transcultural situations which are experienced as demanding or challenging.

### 3.3. How do You Manage Your Work? (Manageability)

Participants refer to three major categories which are most helpful to manage and lead across cultures: positive behaviour, positive attitude and positive emotions (Table 3).

Building an interpersonal connection is part of managing relationships across cultures. This connection should be built on CL and can support coping with challenging transcultural work situations. P1, a German, female leader, says:

“Love gives me the strengths to build bridges and connect to other people, no matter where they come from. I believe in the good of the other and love helps me to do that. Love, for me, is a resource to be together, cooperate and build bridges.”

Generally, respectful behaviour and trust are foundations to manage transcultural and global work relationships (8 participants). P11, a German male leader, highlights:

“When we behave in a loving way, respectful and kind, it is easy to cooperate in a diverse workforce. Then it is really enjoyable.”

Further, eight individuals use knowledge from previous transcultural experiences in which solutions were found. Another 13 participants emphasise that to manage transcultural and global cooperation, they have to understand, respect and value others from different backgrounds. P12, a male Black African leader, highlights both aspects in his response to managing global work relationships:

“We must always see to learn as much as the own and other culture. When we use all this knowledge of humankind in a loving way, we will collaborate well and in a good spirit.”

Finally, five leaders mention that a general love for humanity, a kind of world love, which is often called “agape” is important to manage transcultural situations and individuals. 

With regard to positive behaviour, open communication (7), showing compassion (3) through behaviour and verbally challenging prejudices and racism (3) are mentioned.

Leaders expressed different views on how to manage global work relationships. In total, 13 participants feel that they need to focus on positive emotions to cope with transcultural cooperation. P4, a male German leader, says:

“When you feel positive feeling towards the other it is so much easier to respect, to appreciate and to learn from the other. When I focus on the positive feelings, I can cope much easier with any transcultural situation.”

The majority of leaders found coping easier when positive thoughts, feelings and behaviours were executed or experienced.

### 3.4. What Makes Your Work Meaningful? (Meaningfulness)

Altogether, 51 statements were made with regard to meaningfulness. Many statements refer to meaningfulness and its creation through loving (work) relationships (Table 4). Meaningfulness is strongly constituted through a loving human connection (13 participants). CL represents an overall meaning for 11 participants. This experienced meaningfulness is connected to fulfilment, vocation and life purpose. The following statements refer to CL and meaning in this regard. However, four statements also refer to the negative aspects in the case of an absence of CL, which is associated with negative health and depression. P6, a US-American male leader, points out:

“I can devote myself to meaningful work because I have the security that comes with being embedded in relationships of mutual love. And even though I have collaborated with other scholars who I do not particularly know, or sometimes do not know at all, most of my collaborations have been in what I would call loving relationships. And the work we have done or are doing is meaningful both because we are jointly putting our knowledge and abilities to the project and because we like working with one another.”

P18, a female US-American leader, states:

“I have worked for institutions that see their employees and students as fungible cogs in a wheel. These employers use their employees only for the monetary value that can be gained, as if the only value in an institution of higher learning is the bottom line. I am now in a place that recognizes the importance of vocation—both with the faculty and staff and with the students.”

P7, a German female leader, emphasises:

“Love is an energy that transforms people for the better, for peace, sustainability, and humanity, and positivity in its original form.”

CL is viewed as an overall positive concept that is only negative when it is intentionally or unintentionally misused or absent. It is seen as a universal positive source which can connect individuals of different cultures.

### 3.5. Coping Mechanisms

In terms of C mechanisms at work, the participants point out that CL builds a base for salutogenesis and is a major general C mechanism (Table 5). Altogether, 10 statements refer to the idea that loving one’s work and working with people creates happiness and well-being, and thereby acts as a strong social C mechanism. P1, a female German leader, says:

“Working within loving relationships makes me happy.”

An absence of love results in poor health. P3, a male, South African leader, states the following:

“Love relationships impact strongly on my mental health and well-being. I am loved by my family; thus I feel loved and are very happy. However, my organisation is not always in love with me, which impact strongly on my well-being at work. I am not loved by my organisation due to my race, and political agendas. New policies are now recommended that white people not be considered for promotion, to advance black people. This impacts very negatively on my sentiments for my organisation.”

For this leader, the organisation shows love if it acts non-discriminatory. However, this leader does not feel loved because he is structurally discriminated against with regard to his race. This structural discrimination in South Africa is founded in compensation policies and mechanisms stemming from socio-historical discrimination. Therefore, the attitude of this leader could be judged as “non-loving” in a way, because he does not appreciate that the policies are made to compensate for previous injustice. He only sees the policy as being non-loving, since advantages are given to members of other race groups in this case.

Seven individuals view CL as an intra-personal resource—a self-coping mechanism. P22, a German female leader, emphasises:

“There is a close connection between love and mental health. Without love—not to love and to feel, not to be loved—makes you sick. Persons get isolated. But for mental health, it is important, to love yourself to cope with all the challenges. Love is a big resource for myself. It is part of me.”

Further statements regarding CL refer to general CL concepts, seeing CL as a human approach to deal with challenges in life and at work. Again, seven individuals highlight self-love as a key to C, while five people emphasise that loving social support improves mental health. Finally, a few participants view CL as a balancing approach and two people highlight that CL creates a feeling of safety and acts as a coping mechanism.

### 3.6. How Is CL Valued in Your Organisational Leadership Culture?

Leaders provide statements on their views on how CL impacts on their national, socio-cultural and leadership culture. Altogether, 13 individuals say that CL is not a priority in their culture, referring to German, US-American and South African culture explicitly. Four statements mention that love is usually hidden in leadership culture in general. P18, a female, US-American leader, takes a critical view regarding love and her US-American culture:

“In the US love is not valued in leadership culture and barely at all in culture as a whole. Our patriarchal capitalist system is such that it seeks to undermine the individual in order to control her—to summarize Carol Gilligan, patriarchy separates men from women, men from men, and everyone from everyone else. It stunts emotion. There is a false narrative that rationality is the proper focus of attention for a leader, but this rationality rarely is founded in actual logical principles but in a wish to dominate.”

A male, US-American leader, P5, contributes:

“Again, I don’t think most of our political leaders are acting out of tough love, they appear to be acting out of narcissism, which I suppose you could call self-love, or narcissistic love. In America we generally believe that only narcissists are motivated to pursue leadership. My sense is that given the choice, Americans would rather be their own boss, rather than be someone else’s boss and those who aspire to power and rank tend to be those least deserving of it. I suspect this stems from the idealization of the independent family farmer at the time of the US’s initial formation, an ideal that has remained powerful into the 20th century. My ancestors immigrated to rural Minnesota from Europe to farm or start small businesses (barber) and I suspect that small family business ethos continues to permeate the American psyche. Although leaders elsewhere, like Korea, in which hierarchy runs deep through all social relations, do not seem any less narcissistic, loving or noble than anywhere else.”

Ten statements emphasise that CL is of high value in the leadership culture, referring to aspects of CL in the US, German, Black African, Afrikaans and Japanese leadership culture. P9, a German male leader, says that there is CL in the German leadership culture, but there is also a need to increase it:

“I think there can be love in the leadership culture and also one can love their work in the sense of a general love. However, this is rather seldom. We need more love in the workplaces in future to overcome all the challenges.”

Finally, P10, a Japanese female leader, says:

“In our leadership culture, jintokku (benevolence) is valued. Leaders should be competent, but competence only does not necessarily serve as a requirement for a great leader. Of course, leaders should be competent. But at the same time, they have to be kind and considerate towards others.”

According to several participants (Table 6), CL is not necessarily reflected in their national culture or their organisational culture but is often part of their individual or socio-cultural group culture. Several leaders highlight that they would wish for a CL leadership culture and some leaders connect CL with other outstanding values, such as benevolence for the Japanese leadership context.

## 4. Discussion

This study contributes to previous research on salutogenesis and SOC, CL and C in leadership and transcultural and global work contexts [8]. The findings support studies on SOC as a C mechanism to manage work stressors and challenges [9]. The findings show that leaders focus particularly on meaning at work and their lifestyle choices [2], emphasising that CL, as a positive emotion, contributes to managing challenges in contemporary and future workplaces. Leaders in this study do not focus on negative factors when facing challenges during the transition into the 4IR [3] but stay in a positive mindset. This might be a side effect of their strong focus on salutogenesis, SOC, and a strong focus on meaningfulness. They use CL as their main resource for coping at work. The findings, therefore, support previous studies such as Li, Hou and Wu [5] and contribute from a positive psychology mindset to the stress management literature [14]. As shown above [15], CL is defined in the context of three of the six key features [17], namely: listening skills, self-growth, and building a (healthy) community. Leaders in this study foster CL to connect with others, create positive social bonds and grow individually [17].

Meaningfulness is very important for the leaders and provides them with motivation and strengths in 4IR workspaces [20]. SOC and CL can be promoted as new skills and actions to promote systemic, human-based leadership qualities [87], which are important for managing C when faced with new 4IR stressors. The findings do not display a critical view of CL and leadership in relation to Illouz’s [88] critique on capitalist societies. That no critical views on CL are presented might be a shortcoming of the findings. Rather, the participants follow Chandsoda and Salsing’s [26] approach, incorporating sympathy and participating in human relations, based on CL, connecting passionately with the self and other [27,29].

Dooris, Doherty and Orme [36] emphasise that organisations and leaders are increasingly focusing on promoting salutogenesis improve the performance of organisations. The findings show that SOC components can aid leaders [37,38,42]. Further, the findings show that meaningfulness is the most important component, followed by manageability and comprehensibility as SOC, fostering happiness and well-being [46,47], positivity and listening skills [48]. The findings support that CL improves health in leaders, supporting Gray [50]. It is also assumed that CL and SOC are interlinked, including the idea suggested by Eriksson [51] that SOC needs to be applied at different system levels. As in this study, Harry [51] also supports the idea of implementing SOC interventions in organisations. Further, this study supports the idea of fostering positive feelings [63], thereby promoting affection and interest in others [63,64], increasing emotive encounters and meaning [68] through SOC interventions.

As suggested by Winston [89], leaders aim at “doing good” and would like to improve the life of others as well as working “for the better”. Leaders do not refer to negative emotions, such as anger, failure, selfishness, guilt or worry [73]. This positive mindset might increase relaxation, other positive feelings, general well-being [75,76], and spirituality. This further fosters a general appreciation of humanity, with a focus on personal and organisational growth, vocation and professional calling [73]. This study supports previous studies on the positive effects of salutogenesis, SOC and CL regarding C in leadership positions. 

## 5. Limitations

Limitations of this study include the qualitative nature of the research, which only provides a selected, in-depth insight into a small number of participants. These participants are from specific cultures and middle- and higher-economic backgrounds within their societies. The sample is, therefore, biased. The research study provides insights into subjective, ambiguous views and experiences and cannot be generalised [90]. The findings might be helpful to provide a starting point for mixed-method studies on SOC, CL and C.

## 6. Conclusions

The aim of this article was to present the positive coping mechanisms of leaders for coping with challenges faced in the transition into the 4IR with special regard to SOC and CL, thereby contributing to opening a positive and constructive approach on coping during the transition into the 4IR.

Leaders believe in CL as an important mindset to improve transcultural work relationships and to understand complex situations (comprehensibility). CL helps leaders to focus on the positive, look beyond cultural differences and “see the good” in others. CL helps to be appreciative and open-minded and minimises cultural and religious stereotypes. CL relates further to conscious and mindful listening.

Leaders manage transcultural work relationships by primarily focusing on managing behaviour, attitude and emotions (manageability). Healthy workplaces consist of well-managed resources, balanced relationships and positive humane connections. When leaders maintain positive relationships, they cope better with challenges. Love is highly important as a resource to cope with stress.

Meaningfulness is connected with positive and loving relationships, overall meaning creation, purpose and fulfilment, positive feelings, health and well-being. Two individuals view CL and meaningfulness in relation to God.

In conclusion, leaders see a strong connection between CL and C. They feel that CL is strongly associated with being human, happiness, balance and harmony, love for the world and safety. Leaders also highlight that when they have to make decisions, maintaining a CL perspective is often challenging. 

Overall, CL is a strong coping mechanism in leaders of this study. CL is connected with SOC and C (40 statements). CL is a social and intra-personal coping mechanism as well as an important part of the leaders’ personal leadership culture, though not necessarily part of the national leadership culture. CL is a concept that needs to be actively implemented into leadership.

## Figures and Tables

**Table 1:** Connection between love and mental health.

Frequency	Category	Participants
19	Base for well-being	P1, P3, P4, P5, P7, P8, P10, P11, P12, P13, P14, P15, P16, P17, P18, P19, P20, P21, and P22
8	Love is a coping resource	P4, P5, P7, P9, P11, P14, P15, and P22
7	Self-love improves mental health	P1, P7, P10, P12, P14, P21, and P22
7	Love makes us human	P2, P5, P12, P14, P15, P19, and P21
6	Base for happiness	P1, P3, P7, P8, P15, and P20
5	Love creates balance and harmony	P1, P16, P18, P19, and P20
4	Love for the world improves well-being	P1, P5, P7, and P21
2	Base for safety	P3 and P5

**Table 2:** Comprehensibility and love.

Frequency	Overall Category	Category	Participants
Comprehensibility through… (31)			
13	Attitude	Understand transcultural experiences with a loving attitude	P1, P2, P5, P7, P9, P10, P13, P14, P16, P18, P19, P20, and P21
8	Appreciation	Appreciate others across cultures through an open mindset	P1, P6, P12, P18, P12, P8, P16, and P20
7	Learning	Learn about others to increase understanding	P4, P5, P8, P11, P14, P18, and P19
3	Listen	Listen with patience and kindness	P2, P17, and P22

**Table 3:** Manageability and love.

Overall Category	Frequency	Category	Participants
Positive behaviour (33)	9	Build an interpersonal connection	P7, P16, P8, P16, P20 P17, P22, P12, and P20
	8	Be respectful and trusting	P2, P3, P6, P9, P10, P14, P18, and P22
	7	Open communication	P1, P2, P9, P7, P9, P14, and P18
	6	Show compassion through behaviour	P2, P9, P15, P16, P21, and P22
	3	Challenge prejudices and racism (verbally and through actions)	P5, P11, and P20
Positive attitude (26)	13	Actively understand, accept, respect and value the perspective of others	P2, P3, P4, P5, P6, P8, P10, P11, P13, P14, P16, P18, and P22
	8	Use knowledge from different transcultural solutions	P1, P7, P8, P9, P14, P18, P19, and P20
	5	Show love for humanity	P5, P7, P16, P19, and P21
Positive emotions (13)	13	Focus on positive emotions in transcultural cooperation	P1, P7, P9, P10, P13, P18, P19, P20, P21 P2, P5, P14, and P16

**Table 4:** Meaningfulness and love.

Overall Category	Frequency	Category	Participants
Love creates meaningfulness (51)	13	Meaningfulness creation through loving work relationships and social connection at work, transformation of relationships	P4, P5, P6, P7, P8, P10, P11, P13, P15, P18, P19, P20, P21, and P22
	11	Love has an overall meaning	P1, P3, P4, P5, P6, P7, P8, P18, P19, P20, P21, and P22
	10	Love constitutes fulfilment, vocation and purpose in life	P3, P5, P6, P7, P15, P18, P19, P20, P21, and P22
	6	Love creates meaning to know what is good for me	P7, P9, P10, P15, P18, and P19
	5	Love creates health and happiness	P4, P10, P13, P15, and P19
	4	No love brings depression, health risks	P3, P4, P15, and P16
	2	The love to God gives meaning	P19 and P20

**Table 5:** Love as a coping mechanism.

Overall Category	Frequency	Category	Participants
Love as a general coping mechanism (40)	18	My love builds a base for well-being and coping with challenges	P1, P3, P4, P5, P7, P8, P10, P11, P12, P13, P14, P15, P16, P17, P18, P19, P20, and P21
	8	Love is a resource	P4, P5, P7, P9, P11, P14, P15, and P22
	7	Love is a human approach	P2, P5, P12, P14 P15, P19, and P21
	5	Love creates balance	P16, P19, P20, P1, and P18
	2	Safety	P3 and P5
Love as a social coping mechanism (15)	10	Love my work and working with people is a base for happiness and well-being	P1, P3, P7, P8, P15, P20, P1, P5, P7, and P21
	5	Loving social support improves mental health	P8, P15, P19, P20, and P22
Self-love as a coping mechanism	7	Self-love improves mental health	P1, P7, P10, P12, P14, P21, and P22

**Table 6:** Love in leadership culture.

Overall Category	Frequency	Category	Participants
Love is not a priority in leadership culture (13)	5	In German culture—the word love is not mentioned in German leadership culture	P4, P6, P7, P8, P13, P16, P19, and P22
	4	Generally hidden in leadership culture	P3, P4, P8, and P11
	3	In US culture	P5, P16, and P18
	1	In South African culture	P14
High value of love… (10)	5	In US culture, through understanding and freedom and “tough love”	P5, P6, P11, P12, and P16
	2	German leadership culture	P9 and P16
	1	Black African culture	P12
	1	Afrikaans culture	P3
	1	Japan: Jintokku (benevolence)	P10

## Appendix A

Biographical data

Sex:

Age:

Mother tongue:

Cultural background:

Nationality:

Religious affiliation/belief:

Highest educational degree:

Profession:

Leadership position:

Marital status:

Marital status with someone from another culture? If yes, which cultures:

Have you have lived outside of your birth country (more than one year)?

If yes, where did you live longer than one year?

1. What is love for you and how to you show love in professional contexts?
2. Please give an example of love in the workplace.
3. How are love and leadership connected?
4. How does love impact on your (work) relationships?
5. How can love support the establishment of positive transcultural interaction?
6. How are love and mental health and well-being connected?
7. How do you understand the world?
8. What is important to understand the world?
9. Which resources do you use to cope with challenging work situations?
10. What makes your life and your work meaningful?
11. Which resources to you use to cope with the transformation into the 4IR?
12. How is love valued in your culture/in your leadership culture. Please give an example.

## References

[1] Philbeck T., Davis N. (2019). The fourth industrial revolution: Shaping a new era. J. Int. Aff..

[2] Fink A., Elisabetta E. (2019). Skill flows and the Fourth Industrial Revolution: Future questions and directions for the ASEAN Economic Community. Skilled Labour Mobility and Migration.

[3] Schwab K. (2017). The Fourth Industrial Revolution.

[4] Kamaruddin S.F., Laura D.I., Susan R.J., Musa B.M., Eng T.H. (2019). The impact of listening and speaking anxieties on the fourth Industrial revolution: What can educators do?. J. Lang. Commun..

[5] Li G., Hou Y., Wu A. (2017). Fourth Industrial Revolution: Technological drivers, impacts and coping methods. Chin. Geogr. Sci..

[6] Antonovsky A. (1979). Health, Stress and Coping.

[7] Antonovsky A. (1987). Unravelling the Mystery of Health: How People Manage Stress and Stay Well.

[8] Bauer G.F., Roy M., Bakibinga P., Contu P., Downe S., Eriksson M., Mana A. (2019). Future directions for the concept of salutogenesis: A position article. Health Promot. Int.

[9] Saboga-Nunes L., Bittlingmayer U.H., Okan O. (2019). Salutogenesis and health literacy: The health promotion simplex. International Handbook of Health Literacy.

[10] Wallace D., Boynton M., Lytle L. (2017). Multilevel analysis exploring the links between stress, depression, and sleep problems among two-year college students. J. Am. Coll. Health.

[11] Maxwell J.S., Davidson R.J. (2007). Emotion as motion: Asymmetries in approach and avoidant actions. Psychol. Sci..

[12] Maykrantz S.A., Houghton J.D. (2020). Self-leadership and stress among college students: Examining the moderating role of coping skills. J. Am. Coll. Health.

[13] Shuck B., Alagaraja M., Immekus J., Cumberland D.M., Honeycutt-Elliott M. (2016). Operationalizing Compassionate Leadership Behavior. Academy of Management Proceedings.

[14] Dierendonck D., Patterson K. (2015). Compassionate love as a cornerstone of servant leadership: An integration of previous theorizing and research. J. Bus. Ethics.

[15] Reynolds K. Is Servant-Leadership Gender Bound in the Political Arena?. Proceedings of the Annual Meeting of the Midwest Political Science Association 67th Annual National Conference.

[16] Reynolds K. (2015). Servant-Leadership—A Feminist Perspective. Int. J. Servant Leadersh..

[17] Villiers C. (2019). Boardroom Culture: An Argument for Compassionate Leadership. Eur. Bus. Law Rev..

[18] De Zulueta P.C. (2016). Developing compassionate leadership in health care: An integrative review. J. Healthc. Leadersh..

[19] Lloyd J.L. (2017). Relationship between Self-Compassion, Sense of Coherence, Coping Strategies and Perceived Stress in Clinical Psychology Trainees. Ph.D. Thesis.

[20] Alabi M., Telukdarie A., Van Rensburg N. Industry 4.0: Innovative solutions for the water industry. Proceedings of the International Annual Conference of the American Society for Engineering Management.

[21] Sisodia R., Wolfe D., Sheth J.N. (2003). Firms of Endearment: How World-Class Companies Profit from Passion and Purpose.

[22] World Economic Forum (2016). The Future of Jobs. Employment, Skills and Workforce Strategy for the Fourth Industrial Revolution. Global Challenge Insight Report. http://www3.weforum.org/docs/WEF_Future_of_Jobs.pdf.

[23] Magubane K. (2019). This Love Thing: A New Age Love Story.

[24] Illouz E. (2011). Warum Liebe Weh Tut.

[25] Illouz E. Warum endet die Liebe? Sternstunde Philosophie SRF Kultur. 29 March 2019. https://www.youtube.com/watch?v=3HT3c0rawI0.

[26] Chandsoda S., Salsing P.S. (2018). Compassion and cooperation: The two challenging ethical perspectives in the Fourth Industrial Revolution. J. Int. Bus. Stud..

[27] Van der Hoven J. Using Flow to Create Meaningful Work in the Fourth Industrial Revolution. 22 October 2017. https://leaderless.co/blog/2017/10/22/the-flow-of-the-fourth-industrial-revolution/.

[28] Tu T.N., Thien T.D. (2019). Buddhism and the Fourth Industrial Revolution.

[29] Sun Z. (2018). Innovation and Entrepreneurship in the 4th Industrial Revolution.

[30] Fabritius S. (2017). Ventures for a Better Society; 4th Entrepreneurial Revolution. Master’s Thesis.

[31] Champeau. M., Arsac M., Pineau P., Denyset F. (2016). Non-Standard Employment around the World.

[32] Jae R.N. (2005). Research on the employment instability and its causes. J. Labour Econ..

[33] De Witte H., Pienaar J., De Cuyper N. (2016). Review of 30 years of longitudinal studies on the association between job insecurity and health and well-being: Is there causal evidence?. Aust. Psychol..

[34] Patel P.C., Devaraj S.S., Hicks M.J., Wornell E.J. (2018). County-level job automation risk and health: Evidence from the United States. Soc. Sci. Med..

[35] Rojas-Rueda D. (2017). Autonomous vehicles and mental health. J. Urban Ment. Health.

[36] Dooris M., Doherty S., Orme J., Mittelmark M.B., Sagy S., Eriksson M., Bauer G.F., Pelikan J.M., Lindström B., Espnes G.A. (2017). The application of salutogenesis in universities. The Handbook of Salutogenesis.

[37] Gimpel C., Von Scheidt C., Jose G., Sonntag U., Stefano G.B., Michalsen A., Esch T. (2014). Changes and interactions of flourishing, mindfulness, sense of coherence, and quality of life in patients of a mind-body medicine outpatient clinic. Forsch. Komplementärmedizin.

[38] Krause D.C., Mayer C.H. (2012). Exploring Mental Health: Theoretical and Empirical Discourses on Salutogenesis.

[39] Mayer C.H., Boness C.M. (2013). Creating Mental Health across Cultures. Coaching and Training for Managers.

[40] Mayer C.H., Krause C., Mayer C.H., Krause C. (2016). Promoting mental health and salutogenesis in transcultural organisational and work contexts. International Review of Psychiatry 23, December 2011.

[41] Mayer C.H., Krause C. (2013). ‘Editorial’, in C.-H. Mayer & C. Krause (eds.), ‘Salutogenese in Beratung und Psychotherapy’ [Salutogenesis in counselling and psychotherapy]. Prax. Klin. Verhalt. Und Rehabil..

[42] Tan K.K., Chan S.W.C., Wan W., Vehviläinen-Jukunen K. (2016). A salutogenic program to enhance sense of coherence and quality of life for older people in the community: A feasibility randomized controlled trial and process evaluation. Patient Educ. Couns..

[43] Mayer C.H., Boness C.M. (2011). Interventions to promoting sense of coherence and transcultural competences in educational contexts. Int. Rev. Psychiatry.

[44] Mayer C.H., Louw L., Von der Ohe H. (2019). Sense of coherence in Chinese and German students. Health Sa Gesondheid.

[45] Pretorius N., Arcelus J., Beecham J., Dawson H., Doherty F., Eisler I., Gallagher C., Gowers S., Isaacs G., Johnson-Sabine E. (2009). Cognitive-behavioural therapy for adolescents with bulimic symptomatology: The acceptability and effectiveness of internet-based delivery. Behav. Res. Ther..

[46] Mayer R.E. (2011). Applying the Science of Learning.

[47] Mitonga-Monga J., Cilliers F. (2016). Perceived ethical leadership: Its moderating influence on employees’ organisational commitment and organisational citizenship behaviours. J. Psychol. Afr..

[48] Mitonga-Monga J., Coetzee M., Cilliers F. (2012). Perceived leadership style and employee participation in a manufacturing company in the Democratic Republic of Congo. Afr. J. Bus. Manag..

[49] Eriksson M. (2017). The Sense of Coherence in the Salutogenic Model of Health. https://www.ncbi.nlm.nih.gov/books/NBK435812/.

[50] Gray D., Alvinius A. (2017). Developing leadership resilience through a sense of coherence. Contemporary Leadership Challenges.

[51] Harry N. (2019). Employee Well-Being in the African Call Centre Digital Workspace. Thriving in Digital Workspaces.

[52] Konstam V., Celen-Demirtas S., Tomek S., Sweeney K. (2015). Career adaptability and subjective wellbeing in unemployed emerging adults: A promising and cautionary tale. J. Career Dev..

[53] Gross J.J. (1998). Antecedent- and response-focused emotion regulation: Divergent consequences for experience, expression, and physiology. J. Personal. Soc. Psychol..

[54] Segerstrom S.C., Smith G.T. (2019). Personality and coping: Individual differences in responses to emotion. Annu. Rev. Psychol..

[55] Bossuyt E., Moors A., DeHouwer J. (2014). On angry approach and fearful avoidance: The goal-dependent nature of emotional approach and avoidance tendencies. J. Exp. Soc. Psychol..

[56] Machin J.E., Adkins N.R., Crosby E., Farrell J.R., Mirabito A.M. (2019). The marketplace, mental well-being, and me: Exploring self-efficacy, self-esteem, and self-compassion in consumer coping. J. Bus. Res..

[57] Folkman S., Moskowitz J.T. (2004). Coping: Pitfalls and promise. Annu. Rev. Psychol..

[58] Hoyle J.R. (2001). Leadership and the Force of Love. Six Keys to Motivating with Love.

[59] Barsade S.G., Gibson D.E. (2007). Why does affect matter in organizations?. Acad. Manag. Perspect..

[60] Barsade S., Brief A.P., Spataro S.E., Greenberg J., Mahwah N.J. (2003). The affective revolution in organizational behavior: The emergence of a paradigm. Organizational Behavior: The State of the Science.

[61] Hareli S., Rafaeli A., Brief A.P., Staw B.M. (2008). Emotion cycles: On the social influence of emotion in organizations. Research in Organizational Behaviour.

[62] Robinson M.D., Watkins E.R., Harmon-Jones E. (2013). Handbook of Cognition and Emotion.

[63] Eldor L. (2017). Public Service Sector: The compassionate workplace—The effect of compassion and stress on employee engagement, burnout, and performance. J. Public Adm. Res. Theory.

[64] Underwood L.G., Post S.G., Underwood L.G., Schloss J., Hurlbut W.B. (2009). The human experience of compassionate love. Altruism and Altruistic Love.

[65] Patterson K., van Dierendonck D., Patterson K. (2010). Servant Leadership and Love. Servant Leadership.

[66] Barsade S.G., O’Neill O.A. (2014). What’s love got to do with it? A longitudinal study of the culture of companionate love and employee and client outcomes in a long-term care setting. Admin. Sci. Q..

[67] Fineman S. (2000). Emotions in Organizations.

[68] Savickas M.L. (1991). The meaning for work and love: Career issues and interventions. Career Dev. Q..

[69] O’Neill O.M. (2018). The FACCTs of (work) life: How relationships (and returns) are linked to the emotional culture of companionate love. Am. J. Health Promot..

[70] Rynes S.I., Bartunek J.M., Dutton J.E., Margolis J.D. (2012). Care and compassion through an organizational lens: Opening up new possibilities. Acad. Manag. Rev..

[71] Parry K., Kempster S. (2013). Love and leadership: Constructing follower narrative identities of charismatic leadership. Manag. Learn..

[72] Rapport N. (2019). Cosmopolitan Love and Individuality: Ethical Engagement beyond Culture.

[73] Fry L.W., Matherly L.L. Spiritual Leadership and Organizational Performance: An Explorative Study. Proceedings of the Academy of Management Meeting.

[74] Soh C., Connolly D. (2020). New Frontiers of Profit and Risk. The Fourth Industrial Revolution’s Impact on Business and Human Rights. New Political Econ..

[75] Cooper M. (2013). The Compassionate Mind Approach to Reducing Stress.

[76] Gilbert P. (2009). The compassionate Mind: A New Approach to Life’s Challenges.

[77] Creswell W. (2013). Qualitative Inquiry and Research Design: Choosing among Five Approaches.

[78] Hassan I., Ghauri P.N. (2014). Research Design. Int. Bus. Manag..

[79] Bleicher J. (1980). Hermeneutics as Method, Philosophy and Critique.

[80] Willcocks L.P., Mingers J. (2004). Social Theory and Philosophy for Information Systems.

[81] Naderifar M., Goli H., Ghaljaie F. (2017). Snowball Sampling: A Purposeful Method of Sampling in Qualitative Research. Strides Dev. Med. Educ..

[82] Rahi S. (2017). Research design and methods: A systematic review of research paradigms, sampling issues and instruments development. Int. J. Econ. Manag. Sci..

[83] Terre Blanche M., Durrheim K., Painter D. (2006). Research in Practice: Applied Methods for the Social.

[84] Yin R.K. (2014). Case Study Research: Design and Methods.

[85] Colepicolo E. (2015). Information Reliability for Academic Research: Review and Recommendations.

[86] Roth W.M., Unger H.V. (2018). Current Perspectives on Research Ethics in Qualitative Research. Forum Qual. Soz. Forum Qual. Soc. Res..

[87] Mayer C.H., Coetzee M. (2019). Key factors of Creativity and the Art of Collaboration in Twenty-First-Century workplaces. Thriving in Digital Workplaces: Innovations in Theory, Research and Practice.

[88] Illouz E. (2007). Cold Intimacies: The Making of Emotional Capitalism.

[89] Winston B.E. (2002). Be a Leader for God’s Sake.

[90] Atieno O.P. (2009). An analysis of the strengths and limitation of qualitative and quantitative research paradigms. Probl. Educ. 21st Century.

